# Optimizing plant nutrient acquisition under hypoxia: likely trade-offs, implications for breeders, and lessons from wetland species

**DOI:** 10.1093/jxb/erag093

**Published:** 2026-02-19

**Authors:** Ping Yun, Sergey Shabala

**Affiliations:** School of Biological Sciences and ARC Training Centre for Smart and Sustainable Horticulture, University of Western Australia, Crawley, WA 6009, Australia; School of Biological Sciences and ARC Training Centre for Smart and Sustainable Horticulture, University of Western Australia, Crawley, WA 6009, Australia; College of Plant Science and Technology, Huazhong Agricultural University, Wuhan, China; Nagoya University, Japan

**Keywords:** Flooding, heavy metal toxicity, hypoxia, ion transport, iron, manganese, nitrogen, nutrient acquisition, pyrophosphate, wetland

## Abstract

Hypoxic conditions in waterlogged or flooded soils result in massive penalties to crop production and food security, and the problem is only going to increase under current climate scenarios. While the number of papers dealing with adaptive responses of plants to flooding is increasing exponentially, most staple crops remain highly sensitive to excessive water in the soil. In this work, we analyze the likely reason for this discrepancy. We argue that the current focus on traits aimed to increase the oxygen level in plant tissues under conditions of soil flooding (such as aerenchyma formation, developing adventitious roots, or formation of a radial oxygen loss barrier) is not sufficient to account for all constraints affecting crop performance under stress conditions. By conducting a bioinformatic analysis of a large number of wetland and dryland species, we show that the former species possess a much larger number of gene copies that allow plants to improve acquisition of essential nutrients (such as N and P) as well as to effectively deal with elemental toxicities (e.g. Mn and Fe) originating from changes in redox potential in flooded soils. We then call for a major paradigm shift in our approach to breeding for improved waterlogging stress tolerance by complementing oxygen supply-related traits to those related to vacuolar sequestration of heavy metals and improved nutrient use efficiency. The likely trade-offs of this approach for crop growth under normoxic conditions are discussed.

## Teaser

We undertook a comparative bioinformatic analysis between dryland and wetland species, revealing that a larger proportion of gene copies related to nutrient acquisition may contribute to adaptation of wetland plants under hypoxic soil conditions.

## Introduction

Global climate change increases the frequency and severity of extreme weather events, hindering agricultural production and threatening food security. The natural disasters caused an estimated US$3.8 trillion loss (on average US$123 billion annually) over the period 1991–2021 by impacting agriculture. Flood was the third largest factor, accounting for 16% of agricultural production loss, after extreme temperature (26%) and drought (19%) (FAO, 2023). A recently released flood inundation map showed that the flood risk is widespread and overlaps with croplands across several continents (Fig. 1) (Rogers *et al*., 2025). Both heavy precipitation and flooding can lead to excessive water levels in arable lands (i.e. waterlogged or submerged soil) which causes oxygen (O_2_) deficiency in the soil due to continuous O_2_ consumption (by plants and microbes) and limited O_2_ diffusion from the atmosphere to flooded soil, leading to hypoxia (low O_2_) or anoxia (no O_2_) conditions (Renziehausen *et al*., 2024b).

**Fig. 1.**
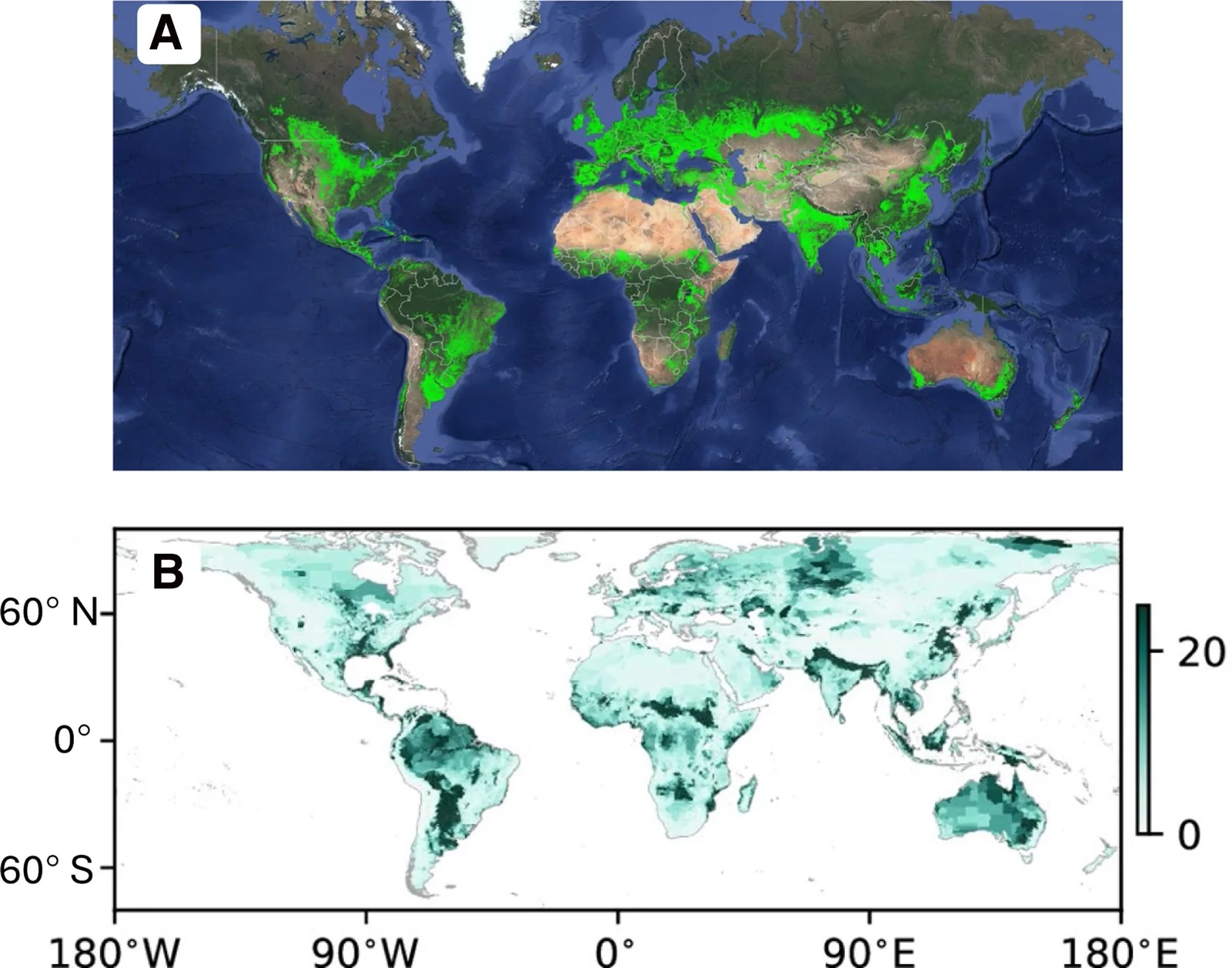
Maps of global cropland and flood inundation area distribution. (A) Map of global cropland distribution (public resource downloaded from U.S. Geological Survey, www.usgs.gov/media/images/map-worldwide-croplands). (B) Map of present-day flood inundation. Reproduced from Rogers *et al*. (2025). The color scale represents the percent inundated area for combined coastal ocean and lake, fluvial, and pluvial flooding.

Abiotic stress tolerance was present in wild relatives of modern crops but then was lost during domestication (Rawat *et al*., 2022; Palmgren and Shabala, 2024). This is also true for tolerance to soil waterlogging/flooding. Of all major staple crops, only rice is classified as a wetland species that can perform well in semi-aquatic conditions. All other major cereals (wheat, maize, and barley), pulses, and horticultural crops are highly sensitive to soil waterlogging. Taking wheat as an example: 2–3 weeks of waterlogging reduced wheat shoot biomass by up to 70% (Malik *et al*., 2002; Herzog *et al*., 2016) while grain yield was reduced by 37–60%, depending on genotype (Alizadeh-Vaskasi *et al*., 2018). This comes with a massive economic cost. On a global scale, soil flooding costs the economy over €60 billion in lost production (Voesenek and Sasidharan, 2013), or about US$88 billion in current prices. As the frequency and severity of flooding events are only going to increase (Fig. 2A), so will the cost. This makes improving waterlogging stress in crops one of the key priorities for breeders. However, despite the exponential growth of papers published on the topic (Fig. 2B), progress in the field is rather modest, given the above penalties on crop yield. So, are we chasing a wild rabbit? Are our breeding programs focused on the right targets? To answer this question, we need to look at the impact of soil flooding on plant growth and the mechanistic basis of plant adaptation to waterlogging stress.

**Fig. 2.**
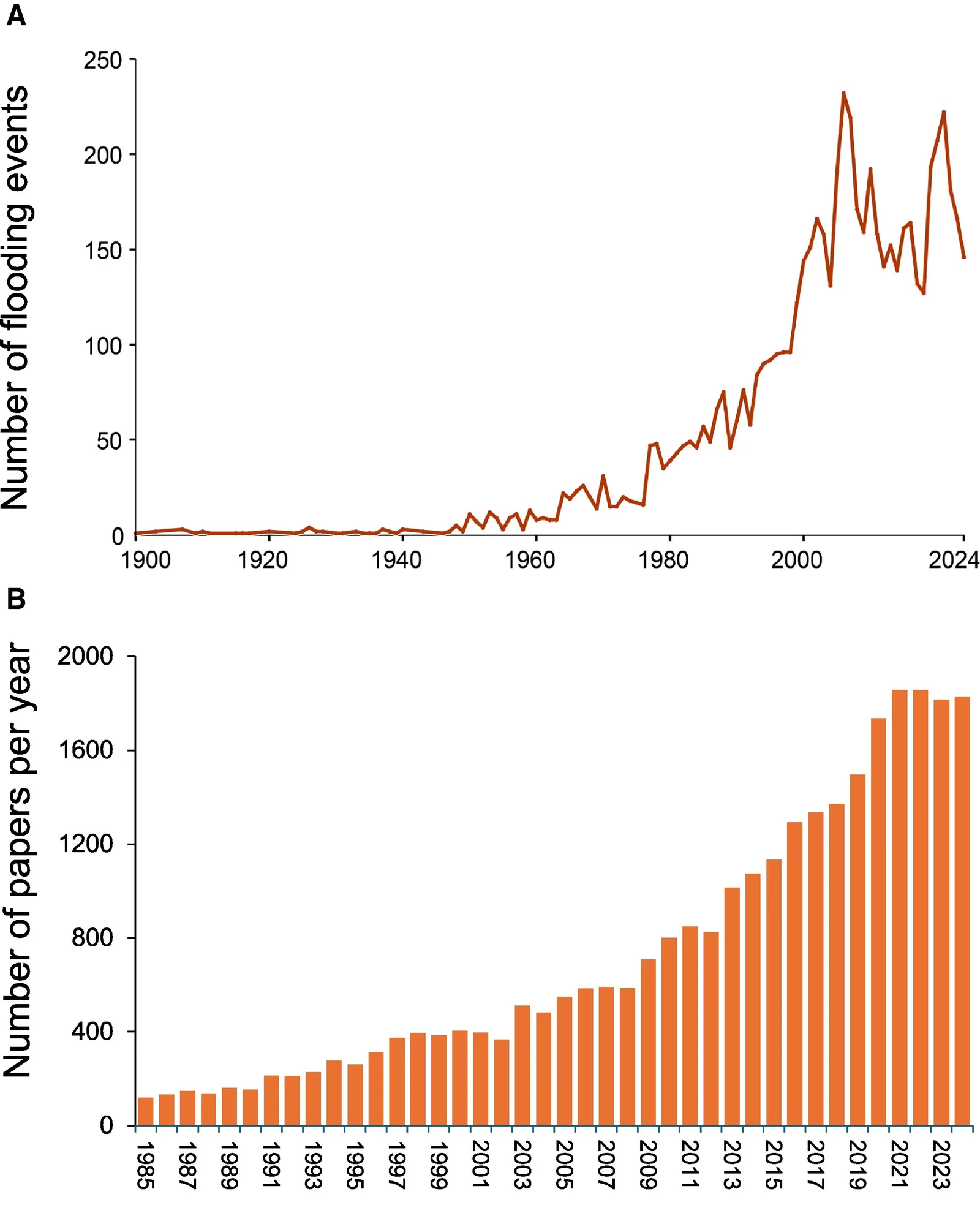
The trend in the number of globally reported flooding events from 1900 to 2024 (A) and exponential increase in the number of papers dealing with the impact of soil flooding on plant performance (B). The data in (A) were obtained from EM-DAT, CRED/UCLouvain (2025)—with major processing by Our World in Data. The latter data are based on a search of the Web of Science using the keywords ‘waterlogging or flooding’ and ‘crop or plant’.

## Hypoxia impacts on plant growth and plant adaptation strategies

Under hypoxia, the root growth of plants is inhibited, and the reduced hydraulic conductance in the root leads to lower shoot hydraulics and leaf water content, inducing stomatal closure and disturbing transpiration (Jackson, 2002; Nicolás *et al*., 2005; Haverroth *et al*., 2024; Kubota *et al*., 2025). Consequently, photosynthesis is constrained by limited CO_2_ supply. Nutrient mobilization, along with transpiration flow, could also be impaired. Plants develop several strategies to adapt to flooding-induced hypoxia (Mommer *et al*., 2007; Colmer and Voesenek, 2009; Pedersen *et al*., 2021; Daniel and Hartman, 2024; Peralta Ogorek *et al*., 2024).

For the root, responses to flooding involve changes in the root system architecture (RSA) and root anatomy. In dryland species, the growth of the seminal root usually ceases, and most of the monocots and some of the dicots develop adventitious roots from the shoot base and grow close to the soil surface or floating in water. For dryland species that adapted to flooding, such as rice, and for wetland species, adventitious roots also emerge from underwater nodes in the case of (partial) submergence. Lateral root development is stalled, and root growth direction is also altered in response to flooding (Pedersen *et al*., 2021; Daniel and Hartman, 2024). These changes are partially attributed to sensing of hypoxia and modulation of hormonal signaling (Jung and McCouch, 2013; Maurel and Nacry, 2020). Group VII ethylene-responsive factors (ERFVIIs) play a key role as O_2_ sensors in plant cells in the hypoxia response (Licausi *et al*., 2011; Zubrycka *et al*., 2023). Hypoxia is perceived via stabilized ERFVII (reduced oxidation due to low O_2_)-mediated activation of hypoxia-responsive genes (HRGs) (Swain *et al*., 2024). Recent studies showed that both energy (ATP) sensing via target of rapamycin (TOR) and Ca signaling (calcium-dependent protein kinase 12, CPK12) pathways phosphorylate ERFVIIs (Fan *et al*., 2023; Kunkowska *et al*., 2023). Anatomically, plants form aerenchyma and a radial oxygen loss (ROL) barrier to adapt to hypoxia-induced changes in O_2_ dynamics in the root. The adventitious root developed under hypoxia has a larger aerenchyma and cortex-to-stele ratio for O_2_ supply (Pedersen *et al*., 2021). The hypoxia-inducible aerenchyma (i.e. lysigenous aerenchyma), such as in wheat, barley, and maize, is formed through programmed cell death (PCD), whereas the constitutive aerenchyma (i.e. schizogenous aerenchyma), such as in wetland species, is formed by differential cell separation regardless of flooding (Evans, 2004; Yamauchi and Nakazono, 2022). Some dryland dicots, such as soybean, also form secondary aerenchyma through the activity of secondary meristem under flooding, which is porous tissue developed centrifugally from phellogen and distributed in nodules, stems, hypocotyls, and adventitious roots for O_2_ supply (Yamauchi *et al*., 2013). A higher proportion of aerenchyma in the root cortex allows plants to store O_2_ for maintaining active metabolism. Another adaptive feature allowing plants to store a sufficient amount of O_2_ in the root is the formation of a barrier to prevent ROL (Y.-Q. Gao *et al*., 2024; Peralta Ogorek *et al.*, 2024). Such a ROL barrier is achieved by suberization of apoplastic areas of exodermis at the basal area of the root for sustained longitudinal O_2_ diffusion to the root tip, such as in rice (Colmer, 2003).

Plants also adjust shoot growth to adapt to flooding. The first step in this process is stress sensing by roots (Jackson, 2002; Ashikari *et al*., 2025). Hypoxia induces ethylene enrichment in roots by enhancing the biosynthesis of 1-aminocyclopropane-1-carboxylic acid (ACC; the ethylene precursor), and the signal can be transported to the shoot via the xylem (Jackson, 2002). Two strategies for shoot growth could be employed by plants in response to submerged flooding: escaping or quiescence (Bailey-Serres *et al*., 2012). The escaping strategy accounts for elongation growth (internodes or petioles), which in rice is facilitated by the ethylene–gibberellic acid (GA) pathway (Ashikari *et al*., 2025), and in the wetland species *Nasturtium officinale* is driven by abscisic acid (ABA) reduction (Müller *et al*., 2021). Growth quiescence occurs during deep submergence or flash flooding. This response is considered true tolerance to flooding as it limits respiration and reserves carbohydrate, known to be regulated by ethylene-induced *submergence 1* (*SUB1*) in rice (Xu *et al*., 2006; Bailey-Serres and Voesenek, 2008).

At the cellular level, low O_2_ supply limits the energy pool (ATP) produced by respiration (Cho *et al*., 2021; Weits *et al*., 2021). Under normoxia, starch degradation produces hexose, which is then broken down via glycolysis to pyruvate, releasing some energy (2 ATP). With pyruvate as the substrate, O_2_ serves as the electron acceptor in mitochondrial respiration, a process that releases most of the energy (25 ATP from two pyruvates) originally derived from glucose. In cases of O_2_ deficiency, fermentation replaces mitochondrial respiration, producing ATP at much lower efficiency (2 ATP hypoxically versus 36 ATP aerobically), leading to an energy deficit (Cho *et al*., 2021). A limited energy pool restricts the activity of plasma membrane (PM)-localized H^+^-ATPase, thereby disrupting the proton gradient across the PM and causing PM depolarization, which subsequently affects ion transport (Palmgren, 2001). Plants transduce different signals to alleviate these adverse effects, such as increasing γ-aminobutyric acid (GABA) content to restore the depolarized PM (Wu *et al*., 2021) or stimulating Ca signaling via calcium-binding protein families to regulate downstream proteins (Wu *et al*., 2021). Also, cellular nitric oxide (NO) production increases by nitrate reductase and alternative oxidase under O_2_ depletion, and this NO could regulate the activity of many proteins (such as ion transporters) at the post-translational level via *S*-nitrosylation and tyrosine nitration (León *et al*., 2021; Gupta *et al*., 2022; Sandalio *et al*., 2023; Swain *et al*., 2024).

## Is targeting traits enabling plants access to oxygen sufficient to improve flood tolerance?

As commented above, despite an exponential growth of studies on plant adaptation to flooding and significant progress on molecular mechanisms of sensing and adaptation to low O_2_ in plants, flood-tolerant cultivars are still far from being available for farmers’ fields. One of the reasons for this is that the majority of studies on flooding use artificial experimental systems that overlook many factors present in actual flooding conditions (Daniel and Hartman, 2024). In addition to reducing O_2_ availability to plants, waterlogging stress also imposes many other constraints on plants. When plants are grown in the field, flooding-induced hypoxia affects microbial activities in the soil (Lu *et al*., 2024) as well as leading to complex alterations in soil properties (Ponnamperuma and Brady, 1972; Zhang and Furman, 2021). In turn, microbes could trigger toxicity by mobilizing metals through reduction; for example, reduction of Fe(III)/Mn(IV) oxides releases Fe^2+^/Mn^2+^ into water (Myers and Nealson, 1988; Kostka *et al.*, 2002; Chen *et al.*, 2020), and arsenic(V) reduction caused by microbial Fe(III) reduction releases As(III) to soil solutions (Xue *et al*., 2020; A.X. Gao *et al*., 2024). Taken together, this has a massive impact on plant ionic homeostasis and its ability to acquire essential nutrients, and may also result in a range of elemental and metabolic toxicities. These constraints cannot be completely eliminated by improved uptake/storage of O_2_ in the root tissues. Some supporting arguments are given below.

Even if plants possess sufficient amounts of O_2_ (e.g. stored in aerenchyma), prolonged soil waterlogging will lead to a significant decline in the soil redox potential. As shown in Fig. 3, the reduction process of oxidants in the soil generally occurs in the following order: NO_3_^−^ to N_2_, MnO_2_ to Mn^2+^, Fe^3+^ to Fe^2+^, SO_4_^2−^ to H_2_S, and CO_2_ to CH_4_ (Marschner, 2021). After O_2_ is depleted by aerobic respiration of the soil microbiome, anaerobic metabolism will take over and first reduce nitrate to nitrous oxide and N_2_ via denitrification (Kaur *et al*., 2020). In addition, nitrogen removal in the soil also occurs via anammox, converting NO_2_^−^ and ammonium to N_2_. As a result, biological N availability for plants is reduced, with consequences for their growth and metabolism.

**Fig. 3.**
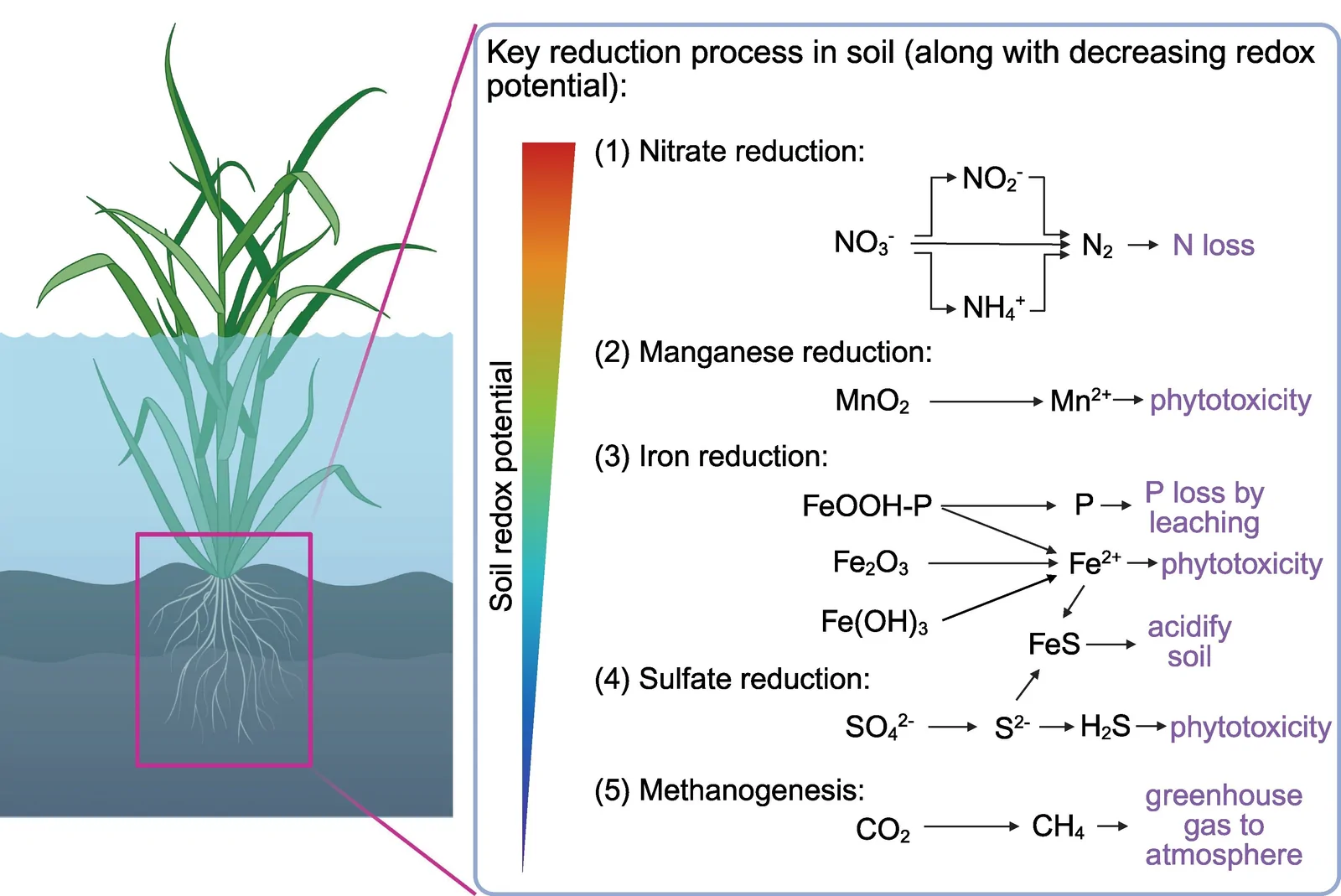
A schematic figure to show the key reduction processes in soil along with decreasing redox potential (triggered by hypoxia) and potential consequences (highlighted with a purple color). The color scale (from red to blue) indicates that the soil redox potential decreases with extension of the flooding period. Reduction processes happen in numerical order as the redox potential declines. Created in BioRender. Yun, P. (2026) https://BioRender.com/w17a120.

When the redox potential becomes lower, Mn^4+^ and Fe^3+^ become electron acceptors, which solubilize manganese oxides and iron oxides, and increase Mn^2+^ and Fe^2+^ content in flooded soil over time to the level toxic to plants (Shabala *et al*., 2014; Wang *et al*., 2022). Thus, in addition to their ability to acquire and accumulate O_2_ for metabolic purposes, plants need to possess mechanisms to deal with accumulation of these elemental toxins, to adapt to flooded soils. Furthermore, under hypoxic conditions, the mobility of soluble reactive phosphorus is primarily governed by iron redox cycling (Pan *et al*., 2021). For certain soil types rich in Fe_2_O_3_ and MnO_2_, such as lateritic soil in subtropical areas, this results in elevated Fe-bound P, which is reduced to soluble Fe^2+^ and P, leading to significant P diffusion and P leaching from the soil (Wang *et al*., 2024). If further reduction (longer flooding) occurs, sulfate reduction and methanogenesis occur (Marschner, 2021). The former, together with Fe^2+^, forms Fe sulfides, which can lead to potential soil acidification, and the latter contributes to methane emission from the soil to the atmosphere. Thus, in addition to possessing a high percentage of aerenchyma and developing adventitious roots for improved O_2_ supply, truly tolerant genotypes must possess mechanisms to deal with elemental toxins (Mn, Fe) and possess higher N and P use efficiency, to compensate for their reduced biological availability. This notion is further supported by the bioinformatic analysis of dryland and wetland species, as discussed in the following section.

## Genetic differences between wetland and dryland plants and potential breeding targets related to ion transport

In nature, wetland plants thrive in environments characterized by chronic flooding, which makes them invaluable models for studying adaptation to ionic changes in flooded soil (Pezeshki, 2001). Morphologically and anatomically, wetland species evolved multiple traits for reaeration in tissue, including adventitious root development, aerenchyma formation (including constitutive aerenchyma) in both shoot and root, and the ROL barrier in root (Pezeshki, 2001; McDonald *et al*., 2002; Colmer and Voesenek, 2009), some of which are also employed by dryland species. Apart from O_2_ supply, wetland plants demonstrate a greater capacity to modulate soil redox status (Mueller and Megonigal, 2024), and they can form Fe plaque in roots to sequester and reduce metal concentrations and shoot translocation (Li *et al*., 2017). However, little is known about whether there is a genetic difference between wetland and dryland species that confers better performance of ion homeostasis in the context of flooding-induced soil ionic changes.

To answer this question, we performed a bioinformatic analysis across 26 species, comprising 12 dryland and 14 wetland species (Supplementary Table S1), to compare genomic differences in ion transport associated with flooding tolerance. Published genome data were obtained from Phytozome, NCBI, and the China National Center for Bioinformation (CNCB) databases (Supplementary Table S1). Phylogenetic relationships across the 26 species were analyzed in OrthoFinder (Fig. 4) (Emms and Kelly, 2019). We then compared predicted gene copies of several genes and gene families that related to different processes between wetland and dryland species. During evolution, selection pressure (by stress or humans) could induce gene copy number variations (gCNVs) (Hastings *et al*., 2009; Zhao *et al*., 2023), which influence gene expression patterns and trait variations (Qin *et al*., 2021). Hence, analyses of gCNVs could help us understand their contribution to environmental adaptation in wetland species and provide insights into cultivar breeding for flooding tolerance. To account for the variation in the ploidy, gene number, and genome size of the analyzed species (Supplementary Table S1), we normalized the absolute number of predicted gene copies to the proportion (per mille, ‰) of total genome genes.

**Fig. 4.**
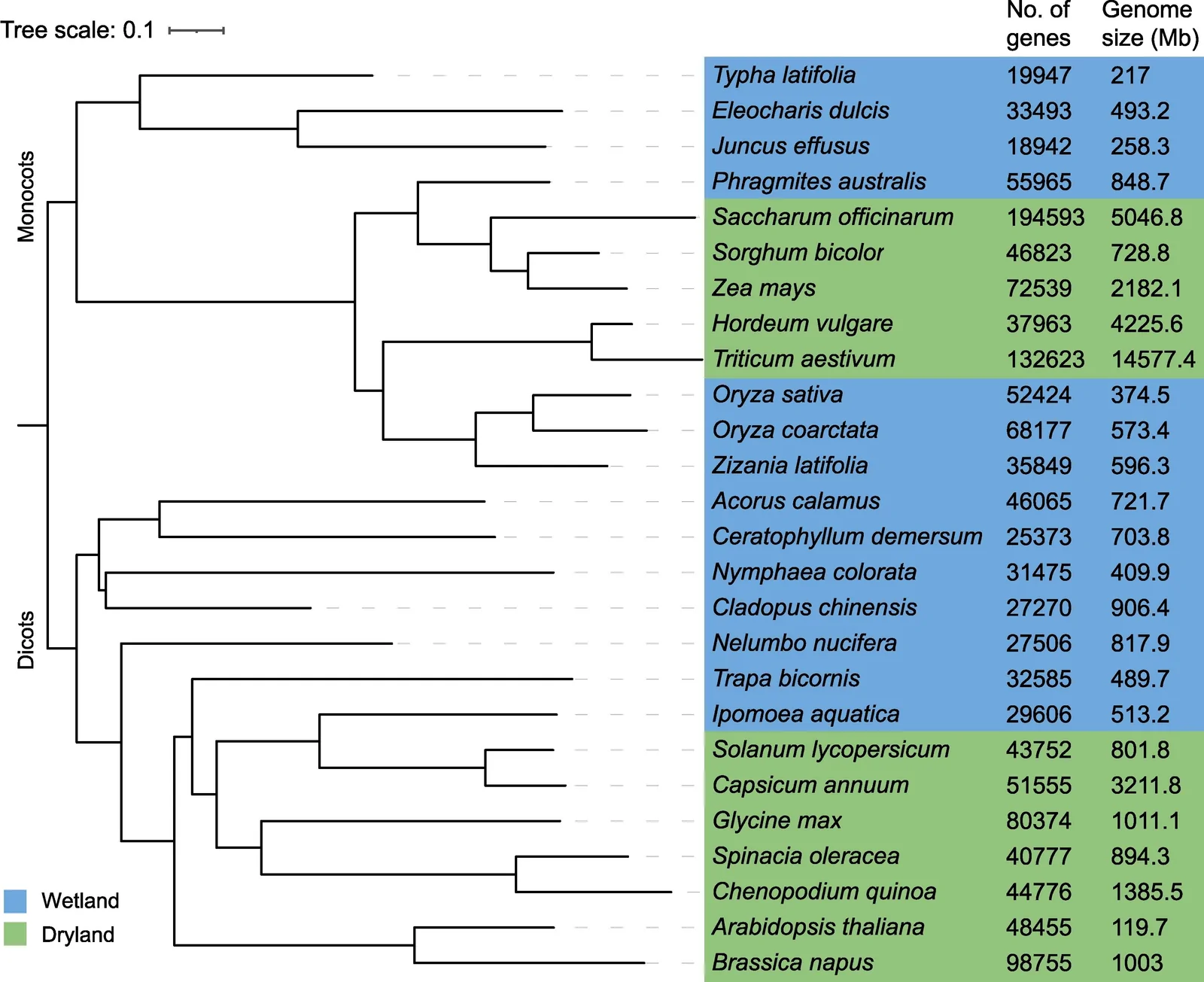
Phylogenetic tree of 14 wetland and 12 dryland species. The analysis was performed in OrthoFinder (Emms and Kelly, 2019) with published genome data (Supplementary Table S1). Prediction for members of gene families uses the Hidden Markov Model (HMM) profile (from Pfam or Panther database) and hmmscan (version 3.4) with default settings. Predictions for genes are extracted from orthogroups of Orthofinder results.

We first checked several genes and gene families involved in ethylene synthesis and signaling (e.g. *ACO1*, the *ACS* family, and the *ERF* family), O_2_ sensing (e.g. *RAP2.12* and *HRE2*), adventitious root formation (e.g. the *MscS* family and the *OSCA* family), and reactive oxygen species (ROS) generation (e.g. the *RBOH* family) (Fig. 5). A rapid elevation in ethylene level is critical for plant response to low O_2_ (Sasidharan and Voesenek, 2015). *In vivo* biosynthesis of ethylene from its direct precursor, ACC, relies on two types of key enzymes, namely ACC synthase (ACS) and ACC oxidase (ACO). The expression of both *ACS* and *ACO* genes is inducible by low O_2_ (Yamauchi and Nakazono, 2022). The ethylene signal is transduced by a group of transcription factors (TFs) called ERFs, which have an ERF domain that directly binds to downstream genes to regulate their expression. The predicted gene copies of *ACO1*, the *ACS* family, and the *ERF* family showed no significant difference between wetland and dryland species in this study (Fig. 5A). Recent studies revealed the key role of ERFVIIs in O_2_ sensing in Arabidopsis, which includes constitutively expressed Related to apetala2.12 (RAP2.12), RAP2.2, and RAP2.3, and hypoxia-up-regulated Hypoxia responsive ERF1 (HRE1) and HRE2 (Zubrycka *et al*., 2023; Swain *et al*., 2024). Under normal conditions, ERFVIIs are degraded by the proteasome, which requires O_2_ and involves the E3 ubiquitin ligase Proteolysis6 (PRT6) in the last step (Licausi *et al*., 2011). In O_2_ deficiency, ERFVIIs are stabilized and can activate hypoxia-responsive genes (Swain *et al*., 2024). There is also evidence showing that O_2_ sensing in plants is K^+^ dependent (Shahzad *et al*., 2016) and regulated by the Ca^2+^ signaling pathway (Bakshi and Gilroy, 2024). Several ion channels, such as Two pore calcium channel 1 (TPC1) and Potassium channel 2 (AKT2), have been proposed as O_2_-sensing candidates based on comparisons with mammalian systems (Wang *et al*., 2017). Here, we examined predicted gene copies of *RAP2.12*, *AKT2*, *PRT6*, *HRE2*, and *TPC1* (Fig. 5B). Notably, *HRE2* and *TPC1* gene copies occupy a larger proportion in the genomes of wetland species compared with dryland species (Fig. 5B). *RAP2.2* and *RAP2.3* are in the same orthogroup as *RAP2.12*, while there is a lack of orthologs of *HRE1* in most species.

**Fig. 5.**
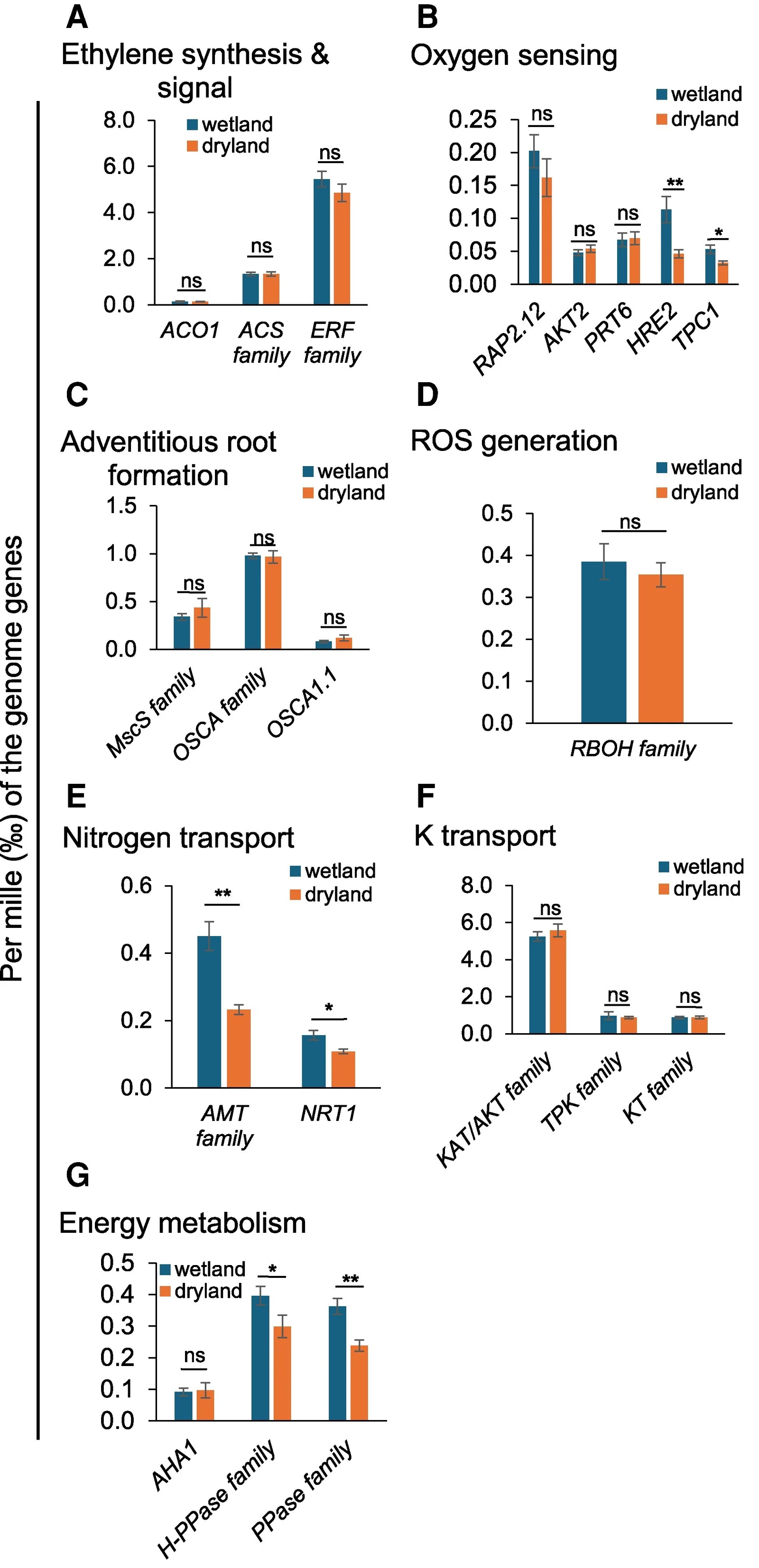
Comparison of predicted gene copies between wetland and dryland species. Genes and gene families that are related to ethylene synthesis and signaling (A), oxygen sensing (B), adventitious root formation (C), reactive oxygen species (ROS) generation (D), nitrogen transport (E), potassium transport (F), and energy metabolism (G). Data are shown as a proportion of genome genes. Predicted gene copies of *ACO1*, *RAP2.12*, *AKT2*, *PRT6*, *HRE2*, *TPC1*, *OSCA1.1*, *NRT1*, and *AHA1* are based on the orthogroups where the following genes are located: *OsACO1*, *AtRAP2.12*, *AtAKT2*, *AtPRT6*, *AtHRE2*, *AtTPC1*, *AtOSCA1.1*, *AtNRT1*, and *AtAHA1*. HMM profiles from Panther (PTHR) or Pfam (PF) were used for predicting gene copies of the *ACS* family (PTHR43795), *ERF* family (PTHR31190), *MscS* family (PF00924), *OSCA* family (PTHR13018), *RBOH* family (PF08414), *AMT* family (PF00909), *KAT/AKT* family (PTHR45743), *TPK* family (PTHR11003), *KT* family (PTHR30540), *H^+^-PPase* family (PF03030), and *PPase* family (PTHR10286). Statistical differences between wetland and dryland species were determined by Student’s *t*-test; *, **, and ns indicate *P*<0.05, *P*<0.01, and not statistically significant, respectively. Details of published genome data can be found in Supplementary Table S1. *ACO1*, *1-aminocyclopropane-1-carboxylate* (*ACC*) *oxidase 1*; *ACS*, *ACC synthase*; *ERF*, *Ethylene responsive factor*; *RAP2.12*, *Related to apetala2.12*; *AKT2*, *Potassium channel AKT2*; *PRT6*, *E3 ubiquitin-protein Proteolysis 6*; *HRE2*, *Hypoxia responsive erf 2*; *TPC1*, *Two pore calcium channel protein 1*; *MscS*, *Small-conductance mechanosensitive channel*; *OSCA*, *Hyperosmolality-gated Ca^2+^ permeable channel*; *RBOH*, *Respiratory burst oxidase homolog protein*; *AMT*, *Ammonium transporter*; *NRT1*, *Nitrate transporter 1.1*; *KAT/AKT*, *potassium channel KAT/AKT*; *TPK*, *two pore domain potassium channel*; *KT*, *Osmotic stress potassium transporter*; *AHA1*, *Plasma membrane-type ATPase 1*; *H-PPase*, *Vacuole-type inorganic H^+^ pyrophosphatase*; *PPase*, *inorganic pyrophosphatase*.

Adventitious root formation is one of the key adaptation traits under flooding. For lateral root development, the mechanical signal could be sensed by ion channels, such as Mechanosensitive ion channel (*MscS*) and Hyperosmolality-gated Ca^2+^-permeable channel (*OSCA*), and transduced into a biological signal (Frachisse *et al*., 2020). Accordingly, predicted gene copies of *MscS* and *OSCA* families and the *OSCA1.1* gene were compared between wetland and dryland plants (Fig. 5C) but no statistical difference was detected. This was the same trend as for the ROS generation gene family, *Respiratory burst oxidase homolog* (*RBOH*) (Fig. 5D). It is unexpected but not surprising that most of the examined genes and gene families (except two in O_2_ sensing) showed a similar proportion of gene copies in the genomes between wetland and dryland species (Fig. 5A–D), as these processes are involved in early flooding adaptation in both types of plants.

In the case of long-term flooding, nitrogen loss and energy crises restrain the growth of dryland species (Cho *et al*., 2021; León *et al*., 2021; Lu *et al*., 2024). We thus asked the question of whether there are any gCNVs in wetland species that may help them accommodate these factors. With this question, we then investigated genes related to nutrient transport (mainly nitrogen and potassium) and energy metabolism (Fig. 5E–G). Ammonium transporters (AMTs) and Nitrate transporters (NRTs) are responsible for the uptake of ammonium and nitrate, respectively (Muratore *et al*., 2021). Interestingly, the predicted gene copies of the *AMT* family and the *NRT1* gene occupy a significantly larger proportion of the genes in the genome in wetland plants than in dryland species (Fig. 5E). Unlike dryland plants such as barley (*Hordeum vulgare*), wetland species demonstrate better tolerance to ammonium toxicity and the capability to utilize ammonium (or even as a preference compared with nitrate) as a nitrogen source for growth, this is seen in paddy rice (Britto *et al*., 2001), *Ceratophyllum demersum* (Best, 1980), *Ipomoea aquatica* (Jampeetong *et al*., 2012), and *Typha latifolia* (Brix *et al*., 2002). Under chronic flooding, the soil nitrogen availability (both NH_4_^+^ and NO_3_^−^) decreases (Kaur *et al*., 2020; Lu *et al*., 2024; Gao *et al*., 2025). Hence, the high proportion of gene copies related to nitrate and ammonium uptake in wetland plants (Fig. 5E) implies that these evolved gCNVs may be attributed to environmental adaptation; vice versa, the gCNVs could contribute to the improved capability to acquire and utilize nitrogen in wetland species under flooded conditions.

Predicted gene copies of the potassium transport KAT/AKT family, TPK family, and KT family were similar between wetland and dryland species (Fig. 5F). Some of them are voltage gated (e.g. AKT/KAT channels) and, hence, operate under strict control of electrogenic H^+^ pumps. The latter are also critical for H^+^-coupled K^+^ symport by high-affinity HAK/KUP transporters (Rubio *et al*., 2020). Under soil flooding, O_2_ deficiency impairs the respiration process and limits the ATP pool, leading to an energy crisis (Colmer and Voesenek, 2009). Here, we examined gCNVs in the PM-localized (P-type) H^+^-ATPase-coding gene *AHA1*, *H^+^ pyrophosphatase* (*H^+^-PPase*), and *PPase* gene families. The predicted gene copies of *AHA1* occupied a similar ratio of the genome in both groups (Fig. 5G), whereas the wetland plants possess a higher proportion of predicted members of *H^+^-PPase* and *PPase* families than dryland species (Fig. 5G). The AHA1 protein uses ATP to pump H^+^ out of the cell; however, when cellular ATP is scarce (such as under flooding conditions), pyrophosphate (PPi) metabolism (via H^+^-PPases or PPases) can be an alternative for energizing the H^+^ gradient across membranes and energy release. In paddy rice, it was proposed that glycolysis occurs in a PPi-dependent manner under hypoxia, a regulation absent in dryland species such as Arabidopsis (Huang *et al*., 2008). It is plausible that the higher ratio of gene copies of *H^+^-PPase* and *PPase* gene families could raise the ceiling of PPi utilization and support wetland species with better energy use efficiency and less ATP dependence, especially under flooding conditions (Igamberdiev and Kleczkowski, 2021).

The reduction in redox potential due to persistent flooding leads to the accumulation of heavy metals (HMs), such as Fe^2+^ and Mn^2+^, in soil. Are the wetland species more tolerant to HM toxicity, compared with their counterparts that grow in dryland environments? Several studies suggest that this is the case (Ait Ali *et al*., 2002; Führs *et al*., 2010; Janeeshma *et al*., 2021). This notion is further supported by our bioinformatic analysis showing a larger proportion of predicted gene copies in genomes of several genes and gene families related to HM transport (Fig. 6). Specifically, gene families of Heavy metal associated (*HMA*), *Cation efflux*, *Zinc transporter* (*ZIP*), and *Copper transporter* (*CPT*) showed a larger proportion of gene copies in genomes of wetland plants than dryland species (Fig. 6A). Also, the predicted gene copies (ratios in the genome) of several genes related to Zn/Mn/Fe transport differ between the two plant groups, including *ZIP1*, *ZIP2*, *Metal tolerance protein* (*MTP8*), *Endoplasmic reticulum* (*ER*)*-type calcium-transporting ATPase 1* (*ECA1*), *ECA3*, *Oligopeptide transporter 3* (*OPT3*), and *Vacuolar iron transporter 1* (*VIT1*) (Fig. 6B–D). Tonoplast-localized ZIP1 and PM-localized ZIP2 are related to Zn/Mn transport in the root, of which ZIP1 relocates Mn/Zn from the vacuole to the cytoplasm and ZIP2 mediates Mn/Zn uptake (Milner *et al*., 2013). On the other hand, tonoplast-localized MTP8, ER-localized ECA1, and Golgi apparatus-localized ECA3 are essential for Mn detoxification by subcellular compartmentation (Wu *et al*., 2002; Mills *et al*., 2008; Chen *et al*., 2013; Takemoto *et al*., 2017). Phloem transporter OPT3 facilitates Fe partition and redistribution (Zhai *et al*., 2014), and tonoplast-localized VIT1 transports Fe^2+^, Zn^2+^, and Mn^2+^ into the vacuole (Zhang *et al*., 2012). The predicted gene copies of all the above HM transport genes have larger proportions in the genome of wetland species (Fig. 6B–D). These results are consistent with the phenotypic evidence of greater HM tolerance in wetland plants. For instance, paddy rice is more tolerant to Mn toxicity than barley, accumulating more Mn without showing toxicity symptoms (Führs *et al*., 2010); reed (*Phragmites australis*) has a higher tolerance threshold to Cu toxicity (78.7 µM) than maize (15.7 µM), and does not suffer from Cu-induced loss of N, P, and K (which happens in maize) (Ait Ali *et al*., 2002); rice also outperforms maize under abundant Zn and Cd conditions with better photosynthetic efficiency (Janeeshma *et al*., 2021). Hence, gCNVs in HM transport genes and gene families may support wetland species in their HM tolerance and acclimation to flooding conditions.

**Fig. 6.**
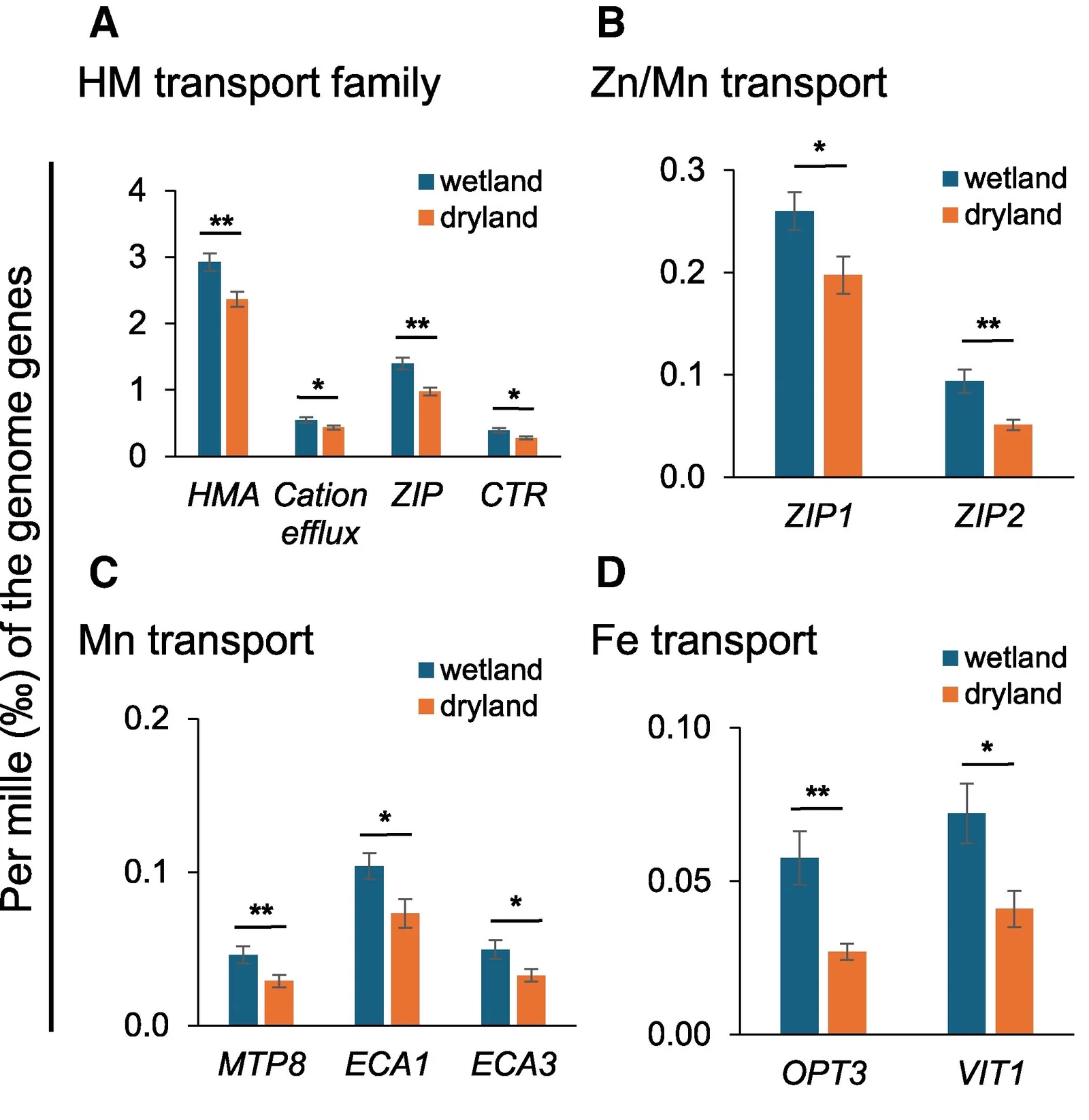
Comparison of predicted gene copies between wetland and dryland species. Genes and gene families that are related to the heavy metal (HM) transport family (A), Zn/Mn transport (B), Mn transport (C), and Fe transport (D). Data are shown as a proportion of genome genes. HMM profiles from Panther (PTHR) or Pfam (PF) were used for predicting gene copies of the *HMA* family (PF00403), *Cation efflux* family (PF01545), *ZIP* family (PF02535), and *CTR* family (PF04145). Predicted gene copies of *ZIP1*, *ZIP2*, *MTP8*, *ECA1*, *ECA3*, *OPT3*, and *VIT1* are based on the orthogroups where the following genes are located: *AtZIP1*, *AtZIP2*, *OsMTP8*, *AtECA1*, *AtECA3*, *AtOPT3*, and *OsVIT1*. Statistical differences between wetland and dryland species were determined by Student’s *t*-test; *, **, and ns indicate *P*<0.05, *P*<0.01, and not statistically significant, respectively. *HMA*, *Heavy metal associated*; *ZIP*, *zinc transporter*; *CTR*, *copper transporter*; *MTP8*, *Metal tolerance protein 8*; *ECA*, *Calcium-transporting ATPase, endoplasmic reticulum-type*; *OPT3*, *Oligopeptide transporter 3*; *VIT1*, *Vacuolar iron transporter 1*.

## Potential trade-offs: impact of the radial oxygen loss barrier on transport of essential nutrients

Dryland cereals (wheat/barley/maize) often exhibit severe nutrient deficiency (N/P/K/Ca/Mg) under flooding conditions due to lack of O_2_ in seminal roots, which consequently reduces the energy pool and slows down operation of H^+^-ATPase, thus hindering the activity of key ion transporters operating in uptake of essential nutrients as well as impacting nutrient loading into xylem and their translocation to the shoot (Colmer and Greenway, 2011). In addition, hypoxic conditions in the rhizosphere lead to a significant depolarization of membrane potential (from −120 mV to −95 mV; Gill *et al*., 2018), making thermodynamically passive nutrient uptake impossible and also leading to a massive loss of essential nutrients through depolarization-activated ion channels. Examples include depolarization-activated PO_4_^−^-permeable channels in lupin root epidermis (Zhang *et al*., 2004), NO_3_^−^-permeable R-type anion channels in Arabidopsis roots (Diatloff *et al*., 2004), and depolarization-activated GORK channels in root epidermis of many species mediating K^+^ loss under stress conditions (Adem *et al*., 2020). Thus, it is not only the inability to acquire enough nutrients due to the lack of O_2_ to fuel H^+^-ATPase but also the inability to retain existing key nutrients (N, P, and K) under conditions of soil flooding that hinders plant performance.

Most wetland species form an effective apoplastic barrier to ROL in the outer cell layers of the root basal zone that, together with aerenchyma, sustains longitudinal O_2_ diffusion to the root tip. The ROL barrier is constitutive in some species and inducible by flooding in others (Colmer, 2003; Pedersen *et al*., 2021; Shiono and Nakazono, 2024). Rice belongs to the latter, and the ROL barrier persists once formed (Colmer, 2003). Formation of the ROL barrier at the root exodermis is attributed to suberization, and suberin biosynthesis-related genes are strongly up-regulated during this process (Shiono *et al*., 2014b; Pedersen *et al*., 2021). Although the regulation governing ROL barrier formation is complex and unclear, studies on rice revealed some essential genes for apoplastic barrier formation. For instance, *Leaf gas film1* (*LGF1*) regulates levels of glycerol esters (a crucial component of suberin lamellae) in exodermal cells, and mutation of *LGF1* reduced glycerol esters and resulted in a weak ROL barrier, while the *LGF1* overexpression line is similar to the wild type (Jiménez *et al*., 2024). As another example, mutation of *Reduced culm number1* (*RCN1*) specifically diminishes suberization of the hypodermis and impairs the ROL barrier in rice, without altering lignin or endodermal suberin in the Casparian strip (Shiono *et al*., 2014a). ABA signaling and low nitrate could induce suberin lamellae formation in the exodermis, thus contributing to the formation of the ROL barrier (Shiono *et al*., 2022, 2024; Zhu *et al*., 2025).

While the main function of the ROL barrier is to prevent O_2_ loss from the root epidermis under hypoxia, this barrier could also physically block the accumulation of unwanted ions or prevent a loss of essential nutrients (Peralta Ogorek *et al*., 2024). For instance, the ROL barrier contributes to improved salt tolerance in the rice variety Pokkali by reducing the amount of accumulated toxic Na^+^ in the shoot (Krishnamurthy *et al*., 2009). In this context, it should be mentioned that many agricultural lands are saline (Liu *et al*., 2020) and, very often, salinity and waterlogging stress can co-occur in nature (Martins *et al*., 2023). Formation of the ROL barrier in rice was shown to be essential for improved tolerance to toxic levels of Fe (Armstrong and Armstrong, 2005) and Cu (Kotula *et al*., 2009). Hence, making the ROL barrier in dryland species constitutive could potentially enhance flooding tolerance. The only issue is a potential impact on the uptake of other essential nutrients. What is the risk, and can it be managed?

Radial uptake of essential nutrients in plants occurs via two parallel pathways—apoplastic and symplastic—and the ROL barrier will only affect the former. The negative impact on symplastic uptake is less obvious. Hence, a decrease in root hydraulic conductivity and delivery of essential nutrients to the shoot following exodermal and endodermal suberin deposition (Peralta Ogorek *et al*., 2024) may be potentially compensated by enhanced symplastic nutrient uptake by manipulating expression levels of high-affinity transporters such as HAK/KUP for K^+^, NRT/NPF for NO_3_^−^, and PHT/PHO for phosphate (Fan *et al*., 2017; Wang *et al*., 2021). The only caveat here is the requirement to energize these (H^+^-coupled) uptake systems (see next section). Also, while modulation of suberin-degrading enzymes in Arabidopsis demonstrated that ectopic suberization in endodermal cells in the *esb1* mutant limits Ca transport and delivery to the shoot (Kamiya *et al*., 2015), it also increased the K content, revealing the complex relationship between the extent of root suberization and selective nutrient uptake/transport.

## Energizing the operation of transporters

One of the most significant effects of O_2_ deficiency in plants is a limitation on energy generation during cellular respiration, reducing ATP production by >90% under hypoxia. The reduced energy pool could consequently restrain ATPase activity. ATPases are a large group of proteins, which can be basically classified into four types: P-type (Palmgren and Nissen, 2011), V-type (Seidel, 2022), F-type (Kühlbrandt, 2019), and ATP-binding cassette (ABC) transporters (Hwang *et al*., 2016). P-type H^+^-ATPases are located at the PM and generate proton and voltage gradients that subsequently drive the transport of ions and nutrients, such as voltage-gated AKT channels for K^+^ uptake (Yuan *et al*., 2024), and H^+^-coupled nitrate (NRT) and phosphate (PHT) transporters (Zhou *et al*., 2021). V-type H^+^-ATPases are located at the tonoplast and control vacuolar ion sequestration and cytosolic pH homeostasis.

The beneficial roles of adaptive morphological traits such as formation of lysigenous aerenchyma or development of the ROL barrier are to a large extent attributed to the ability of a plant to energize H^+^-ATPases by increasing the pool of available ATP. However, given the limited scope of this approach, are there any alternatives to this issue? One such approach is to enhance the anaerobic fermentation process, which consumes more sugar to produce ATP (Summers *et al*., 2000; van Veen *et al*., 2024). Another compensatory approach is to substitute some ATP-dependent processes with PPi-powered alternatives.

It has been suggested that PPi could be an alternative energy source under flooding conditions (Igamberdiev and Kleczkowski, 2021). PPi is a universal byproduct of biosynthesis processes for macromolecules, such as DNA, RNA, proteins, and lipids (Heinonen, 2001). The metabolism of PPi in plant cells is mediated by cytosolic soluble PPase (sPPase) and vacuolar H^+^-PPase (Segami *et al*., 2018). In general, hydrolysis of the high-energy anhydride bond in PPi yields ∼20–30 kJ mol^–1^ of free energy, representing ∼60–80% of that released during ATP hydrolysis (cleavage of the terminal phosphoanhydride), with variance affected by other factors, such as the Mg^2+^ concentration (Alberty, 1969; Rosing and Slater, 1972).

O_2_ depletion almost instantly induces a drop in the cellular ATP level (Saint-Ges *et al*., 1991), whereas the root PPi content appears unaffected by O_2_ deficiency, such as in potato (Mustroph *et al*., 2005) and pea (Dancer and Rees, 1989). In this scenario, Mg^2+^ that is released from ATP and related metabolites in the cytosol could bind to PPi and be utilized by PPi-dependent enzymes, such as PPases (Igamberdiev and Kleczkowski, 2003, 2021). The predicted gene copies of H^+^-PPase and PPase gene families occupy a larger proportion of the genome in wetland species compared with dryland species, and no such difference was found in *AHA1* (Fig. 5C). The gCNVs in PPase families imply that wetland species exploit PPi more, compared with dryland plants, for flooding adaptation. In rice, anoxia induces tonoplast-localized H^+^-PPase activity much more than V-type H^+^-ATPase (75-fold versus 2-fold), suggesting conservation of ATP by using PPi to power H^+^ pumping (Carystinos *et al*., 1995). Moreover, rice up-regulates PPi-dependent reactions, such as pyrophosphate-fructose-6-phosphate-phosphotransferase (PFP) in glycolysis and sucrose breakdown via sucrose synthase, so carbon metabolism keeps running with less ATP cost (Gibbs *et al*., 2000; Fukuda *et al*., 2008; Mustroph *et al*., 2013), whereas such a pathway is largely absent in the dryland species Arabidopsis (Huang *et al*., 2008), indicating that exploiting PPi as an energy source during flooding is feasible. Despite studies showing that overexpression of PPase genes enhances tolerance to salinity, drought, alkali, and cold stresses (Liu *et al*., 2011; Zhang *et al*., 2011; He *et al*., 2017), it remains unknown whether this will also improve flooding tolerance. Nevertheless, transgenic rice with overexpressed Arabidopsis vacuolar H^+^-PPase (*AVP1*) showed higher grain yield under paddy field conditions (Kim *et al*., 2014). Hence, it is plausible that increasing the harnessing of PPi could contribute to flooding tolerance.

The utilization of PPi for enhancing flooding tolerance should not be simply the overexpression of PPases, although this would remove cytosolic PPi. Instead, it should be a coordination of ATPases (P-type and V-type), metabolic PPi production/consumption, and membrane transporters. Otherwise, excess PPase could simply waste PPi or disrupt ion homeostasis, which would also affect plant growth under normal conditions. Another factor that requires caution is post-transcriptional regulation. In Arabidopsis, Ubiquitin-conjugating enzyme34 (UBC34) directly interacts with and ubiquitylates AVP1 to negatively regulate AVP1 activity at the post-translational level (Xu *et al*., 2025). Therefore, manipulation of PPases should also avoid such post-transcriptional suppression. It should be considered that redundant PPi may also be cytotoxic, causing dwarfism, disturbed gluconeogenesis, ectopic starch accumulation, altered cell arrangements, and impaired cell walls (Ferjani *et al*., 2011; Segami *et al*., 2018; Fukuda *et al*., 2020).

## Heavy metal transporters as potential targets for crop improvement for flooded soils

The much larger proportion of predicted gene copies of several genes and gene families related to HM transport in wetland species (Fig. 6) highlights the importance of this trait as a potential target in breeding programs aimed to improve waterlogging stress tolerance in crops.

The HM transport network in plants is sophisticated (Huang *et al*., 2024). Firstly, some transporters are expressed in a tissue-specific manner or with polar localization. For instance, in rice, Yellow stripe-like 2 (OsYSL2) is localized in the phloem companion cells for long-distance Fe transport (Ishimaru *et al*., 2010), and the Natural resistance-associated macrophage protein 5 (OsNRAMP5) and OsMTP9 are respectively localized at the distal and proximal sides of exodermal and endodermal cells for directional uptake of Mn (Huang *et al*., 2024). In addition, many transporters are not exclusive to a single HM, but rather to multiple metals, due to chemical similarity. Overexpression of *Iron regulated transporter 1* (*AtIRT1*) in Arabidopsis leads to not only Fe^2+^, but also Zn^2+^ and Mn^2+^ accumulation (Barberon *et al*., 2011); and OsZIP1 excludes excessive Zn, Cu, and Cd for detoxification in rice (Liu *et al*., 2019). Modifying these transporters could affect the homeostasis of several HMs. Moreover, transporters for essential HMs (Zn, Fe, and Mn) could also be symporters for non-essential HMs (e.g. Cd). The iron transporter AtIRT1 and the Mn transporter OsNRAMP5, which are essential for Fe and Mn uptake, respectively, also mediate Cd uptake (Ishikawa *et al*., 2012; Sasaki *et al*., 2012). OsYSL2 for root-to-shoot Fe transport also translocates Cd (Ishimaru *et al*., 2010). The Zn transporter OsZIP9 takes up Zn and Cd in roots (Tan *et al*., 2020). Mutation of these symporters could reduce the accumulation of Cd, whereas the essential HM uptake or plant growth is also impaired (Ishimaru *et al*., 2010; Ishikawa *et al*., 2012; Sasaki *et al*., 2012; Tan *et al*., 2020). Hence, HM transporter selection should also consider plants that grow in toxic HM-contaminated soil, as flooding may exacerbate the accumulation of unwanted HMs.

Soil chemistry differs between normoxic and hypoxic conditions, especially for Fe and Mn. Both Fe and Mn are essential micronutrients that play vital roles in cellular metabolism. Under normoxic conditions, Fe and Mn deficiency is more common, particularly in calcareous or alkaline soils (White and Broadley, 2009; Alejandro *et al*., 2020). In addition, elevated CO_2_, in the predicted scenario of climate change, leads to reduced Fe and Mn in crops (Yang *et al*., 2024). The biofortification of Fe in edible plant tissues/organs is considered a compensatory approach to alleviate Fe-deficiency malnutrition, especially in developing countries (Bouis and Saltzman, 2017). As a result, the current strategy in crop breeding is to enhance functional expression of transporters mediating uptake and translocation of Fe and Zn—the two most essential micronutrients for human health (Stangoulis and Knez, 2022). However, while this strategy is perfectly justified for crops grown under optimal conditions, it exacerbates the problem of elemental toxicities in flooded soils. The likely compromise may be to combine increased uptake of micronutrients such as Fe, Zn, and Mn under normoxic conditions with their enhanced vacuolar sequestration under soil hypoxia. Some known transporters identified from Arabidopsis or rice include tonoplast-localized AtMTP8, OsVIT1, and AtNRAMP3 (Huang *et al*., 2024).

In general, an ideal combination of traits regarding HM transport to account for flooding tolerance should at least consider the following points: (i) tight control of uptake; (ii) efficient export; and (iii) capability for subcellular sequestration. Tight control of uptake includes enhancing the specificity of HM transporters, at least to discriminate between the uptake of essential HMs and that of toxic HMs. A good example is OsNRAMP3, which not only exclusively transports Mn but also is differentially regulated at low and high Mn concentrations to maintain Mn homeostasis in rice (Yamaji *et al*., 2013). Efficient export involves excluding toxic HMs and excessive essential HMs. For instance, OsZIP1 excludes Cd at low (1 µM) and high (80 µM) Cd concentrations, and it also excludes Zn and Cu but only under high concentrations of Zn and Cu, respectively (Liu *et al*., 2019). In addition, cellular compartmentation is an extra approach for cytoplasmic HM detoxification. For instance, OsMTP8, AtECA1, and AtECA3 partition Mn^2+^ into the vacuole, ER, and Golgi apparatus (Wu *et al*., 2002; Mills *et al*., 2008; Chen *et al*., 2013; Takemoto *et al*., 2017); OsVIT1 sequestrates Fe^2+^, Zn^2+^, and Mn^2+^ into the vacuole (Zhang *et al*., 2012); and OsHMA3 selectively transports Cd into the vacuole (Ueno *et al*., 2010). Nevertheless, for essential HMs, relocation from compartmentation to the cytoplasm for reuse is also necessary, especially under deficiency, as seen with AtZIP1, which releases Mn/Zn from the vacuole to the cytosol (Milner *et al*., 2013). The predicted gene copy ratios of the above-mentioned transporters are higher in wetland plants (Fig. 6), suggesting that, from an evolutionary perspective, they may employ this strategy for flooding adaptation.

Furthermore, coordination of the above components should also be integrated with a finely tuned signaling pathway to balance normoxic and flooding conditions, such as active sequestration only under hypoxic conditions. Such a regulatory network could involve TFs or calcium signaling. Hypoxia intersects with jasmonic acid (JA) (Renziehausen *et al*., 2024a), and the JA-responsive TF MYC2 interacts with MYB43 to suppress the expression of *AtHMA2* and *AtHMA4* by directly binding to their promoters, therefore reducing xylem loading of cadmium (Cao *et al*., 2024). MYC2 could also repress the Fe-like iron-deficiency-induced TF (FIT) network to down-regulate *IRT1* (Cui *et al*., 2018). In Arabidopsis, calcium signaling can stimulate the DNA binding activity of the TF WRKY33 (Zhou *et al*., 2020), which interacts with WRKY12 to up-regulate *AtRAP2.2* (Tang *et al*., 2021) and induce hypoxia responses, playing a key role in hypoxia tolerance. Moreover, WRKY33 could also activate Arabidopsis Tóxicos en Levadura 31 (ATL31) to modulate IRT1 degradation (Zhang *et al*., 2023), which could contribute to less Fe uptake in flooded soil. WRKY33 could also be phosphorylated by Mitogen-activated protein kinase 3 (MPK3) and MPK6 to enhance its transactivation activity (Zhou *et al*., 2020). In addition, MPK3/MPK6 interact and phosphorylate the TF Sensitive to proton rhizotoxicity1 (STOP1), contributing to its accumulation under hypoxia, which controls hypoxia responses in Arabidopsis (Wang *et al*., 2025). STOP1 inhibits the expression of another TF WRKY46 (Tokizawa *et al*., 2023). WRKY46 binds to the promoter of *AtVITL1*, a gene encoding a potential Fe transporter for vacuolar sequestration, to repress its expression (Yan *et al*., 2016). Increased STOP1 under hypoxia could therefore enhance Fe sequestration via VITL1. Nevertheless, all the studies mentioned above are from Arabidopsis, and a similar network in wetland species remains to be explored further.

## Conclusion and future prospects

As discussed above, economic penalties imposed by soil flooding on agricultural crop production are massive, and the acuteness of the problem is only going to increase, given current climate trends. In addition to the cost, it also threatens food security, as all staple crops except rice are highly sensitive to soil flooding. While the extent of the problem is well recognized (Bailey-Serres *et al*., 2025), the breeding efforts lag behind. In our opinion, this is due to the fact that the bulk of the work in this field was focused on the traits related to improved O_2_ supply/accumulation in roots, such as aerenchyma formation or adventitious root development, but largely ignored other constraints present in waterlogged soils. One of them is elemental toxicity. Changes in soil redox potential elevate Mn and Fe concentrations to toxic levels, and increased O_2_ storage capacity in roots cannot prevent this elemental toxicity, unless plants are equipped with efficient tissue tolerance mechanisms. This seems to be the case for wetland species, which have a much higher proportion of gene copies for transporters mediating sequestration of HMs in vacuoles (Fig. 6). However, very few (if any) breeding programs have targeted this trait. In fact, the trend is opposite. Globally, insufficient consumption of micronutrients such as Zn and Fe contributes to nutritional deficiencies in >3 billion people in developing and developed countries, causing serious health concerns related to cognitive development, metabolism, the immune system, obesity, and diabetes (Zhu *et al*., 2018). About half the world’s population have diets too low in Fe (Bouis and Saltzman, 2017), and the problem is not confined to developing countries. Insufficient micronutrient intake is widespread in infants, children, adolescents, and adults, and many diseases associated with aging (cancer, neural deterioration causing mental disorders, etc.) are linked to Fe insufficiency (Benson *et al*., 2025). To fix the problem, for decades cereal breeders have focused on improving the uptake and translocation of Fe to developing grains (Stangoulis and Knez, 2022). While such a strategy is fully justified for crops growing under normoxic conditions, these plants will suffer from Fe toxicity when the soil is flooded.

What can be practical steps? First, we need a paradigm shift from targeting O_2_-related traits to those related to plant tissue tolerance to HMs. A simple search on the Web of Science using keywords ‘aerenchyma’ and ‘QTL’ results in 63 papers published on this topic; this number is even higher (75 papers) for ‘adventitious roots’ and ‘QTL’. At the same time, only eight papers deal with QTLs (quantitative trait loci) for Mn toxicity. The balance is hardly right. We also need to understand how the larger number of isoforms for HM transporters is translated into increased tissue tolerance. A plausible suggestion is that these genes may operate in a highly tissue- and cell-specific manner, thus allowing the organism to diversify its ability to respond to environmental clues. Some subtle differences in the structure of these isoforms may also be expected, modulating their sensitivity to gating signals. For example, Mäser *et al*. (2002) showed that a substitution of glycine by serine in the HKT transporter drastically altered its selectivity and ability to discriminate between Na and K. Also, a mutation from serine to alanine near the gate loop of the PYR1 receptor correlates with differential drought tolerance among evolutionarily diverse species (Chen *et al*., 2024). Finally, the existing conundrum between a need for increased Fe uptake and translocation to the shoot under normoxic conditions and its efficient sequestration in root vacuoles needs to be resolved by understanding the transcriptional regulation and the use of stress-induced TF promoters.

## Supplementary Material

erag093_Supplementary_Data

## Supplementary data

The following supplementary data are available at *JXB* online.

Table S1. Information on the analyzed species.
