# Supplementary material for: Optimizing plant nutrient acquisition under hypoxia: likely trade-offs, implications for breeders, and lessons from wetland species
Source: J Exp Bot. 2026 Feb 19;77(20):6828–43. doi: 10.1093/jxb/erag093 (PMC13720646; doi:10.1093/jxb/erag093)
Supplement: Supplementary file 1 [file erag093_supplementary_data.pdf]

Supplementary Table S1. Information on the analyzed species

No.	Plant type	Taxonomy	Species	Number of genes	Genome size (Mb)	Ploidy level	Reference DOI	URL of genome data
1	wetland	Dicot	*Ceratophyllum demersum*	25373	703.8	polyploid	10.1038/s41477-020-0594-6	https://genomevolution.org/coge/SearchResults.pl?s=56572&p=genome
2	wetland	Dicot	*Cladopus chinensis*	27270	906.4	2	10.1038/s41438-020-0269-5	https://ngdc.cncb.ac.cn/gwh/Assembly/1087/show
3	wetland	Dicot	*Ipomoea aquatica*	29606	513.2	2	10.1016/j.scienta.2023.112193	https://ngdc.cncb.ac.cn/gwh/Assembly/986/show
4	wetland	Dicot	*Nelumbo nucifera*	27506	817.9	2	10.1093/hr/uhad040	https://ngdc.cncb.ac.cn/gwh/Assembly/30224/show
5	wetland	Dicot	*Nymphaea colorata*	31475	409.9	2	10.1038/s41586-019-1852-5	https://ngdc.cncb.ac.cn/gwh/Assembly/651/show
6	wetland	Dicot	*Trapa bicornis*	32585	489.7	2	10.1038/s41597-023-02270-4	https://ngdc.cncb.ac.cn/gwh/Assembly/29371/show
7	wetland	Monocot	*Acorus calamus*	46065	721.7	polyploid	10.1038/s41467-023-38829-3	https://ngdc.cncb.ac.cn/gwh/Assembly/37816/show
8	wetland	Monocot	*Eleocharis dulcis*	33493	493.2	polyploid	10.1038/s41597-024-03717-y	https://ngdc.cncb.ac.cn/gwh/Assembly/83530/show
9	wetland	Monocot	*Juncus effusus*	18942	258.3	2	10.1016/j.cell.2022.06.045	https://www.ncbi.nlm.nih.gov/datasets/genome/GCA_027726005.1/
10	wetland	Monocot	*Oryza coarctata*	68177	573.4	4	10.1038/s41597-023-02594-1	https://ngdc.cncb.ac.cn/gwh/Assembly/37798/show
11	wetland	Monocot	*Oryza sativa*	52424	374.5	2	10.1093/nar/gkl976	https://phytozome-next.jgi.doe.gov/info/Osativa_v7_0
12	wetland	Monocot	*Phragmites australis*	55965	848.7	polyploid	10.12688/wellcomeopenres.23143.1	https://www.ncbi.nlm.nih.gov/datasets/genome/GCF_958298935.1/
13	wetland	Monocot	*Typha latifolia*	19947	217	2	10.1093/g3journal/jkab401	https://ngdc.cncb.ac.cn/gwh/Assembly/37819/show
14	wetland	Monocot	*Zizania latifolia*	35849	596.3	2	10.1038/s41597-025-04853-9	https://ngdc.cncb.ac.cn/gwh/Assembly/88418/show
15	dryland	Dicot	*Arabidopsis thaliana*	48455	119.7	2	10.1111/tpj.13415	https://phytozome-next.jgi.doe.gov/info/Athaliana_Araport11
16	dryland	Dicot	*Brassica napus*	98755	1003	4	10.1038/s41477-019-0577-7	http://cbi.hzau.edu.cn/cgi-bin/rape/download_ext
17	dryland	Dicot	*Capsicum annuum*	51555	3211.8	2	10.1038/s41438-017-0011-0	https://www.ncbi.nlm.nih.gov/datasets/genome/GCF_002878395.1/
18	dryland	Dicot	*Chenopodium quinoa*	44776	1385.5	4	10.1038/nature21370	https://phytozome-next.jgi.doe.gov/info/Cquinoa_v1_0
19	dryland	Dicot	*Glycine max*	80374	1011.1	2	10.1111/tpj.17026	https://phytozome-next.jgi.doe.gov/info/Gmax_Wm82_a6_v1
20	dryland	Dicot	*Solanum lycopersicum*	43752	801.8	2	10.1038/s41586-022-04808-9	https://phytozome-next.jgi.doe.gov/info/Slycopersicum_ITAG5_0
21	dryland	Dicot	*Spinacia oleracea*	40777	894.3	2	10.1038/s41467-021-27432-z	https://www.ncbi.nlm.nih.gov/datasets/genome/GCF_020520425.1/
22	dryland	Monocot	*Hordeum vulgare*	37963	4225.6	2	10.1093/plcell/koab077	https://phytozome-next.jgi.doe.gov/info/HvulgareMorex_V3
23	dryland	Monocot	*Saccharum officinarum*	194593	5046.8	8	10.1038/s41586-024-07231-4	https://phytozome-next.jgi.doe.gov/info/SofficinarumxspontaneumR570_v2_1
24	dryland	Monocot	*Sorghum bicolor*	46823	728.8	2	10.1038/s41597-025-04791-6	https://ngdc.cncb.ac.cn/gwh/Assembly/86159/show
25	dryland	Monocot	*Triticum aestivum*	132623	14577.4	6	10.1111/tpj.15289	https://phytozome-next.jgi.doe.gov/info/Taestivumcv_ChineseSpring_v2_1
26	dryland	Monocot	*Zea mays*	72539	2182.1	2	10.1126/science.abg5289	https://phytozome-next.jgi.doe.gov/info/Zmays_Zm_B73_REFERENCE_NAM_5_0_55
